# From Micro to Macro: A Relativistic Treatment of the Chiral Energy Shifts Caused by Static Electromagnetic Effects on Free Electrons

**DOI:** 10.3390/e24030358

**Published:** 2022-03-01

**Authors:** Philip Kurian

**Affiliations:** 1 Quantum Biology Laboratory, Howard University, Washington, DC 20060, USA; pkurian@howard.edu; 2 Department of Physics and Astronomy, University of Iowa, Iowa City, IA 52242, USA

**Keywords:** Dirac, chiral, quantum field theory, Fröhlich, Zeeman

## Abstract

Free electron systems are ubiquitous in nature and have demonstrated intriguing effects in their collective interactions with weak electric and magnetic fields, especially in aqueous environments. Starting from the Dirac Hamiltonian, a fully relativistic expression is derived for the electron energy shift in the presence of a spatiotemporally constant, weak electromagnetic field. The expectation value of this energy shift is then computed explicitly using the Fourier transforms of the fermionic fields. To first order in the electromagnetic fields, the average relativistic energy shift is found to be completely independent of the electron spin-polarization coefficients. This effect is also considerably larger than that predicted in quantum mechanics by the analogous Zeeman shift. Finally, in the non-relativistic limit, it is shown how to discriminate between achiral and completely polarized states, which leads to a concluding discussion of possible mesoscopic and macroscopic manifestations of electron spin states across many orders of magnitude in the physical world, with stark implications for biological and other complex systems.

## 1. Introduction

Deviations from linear response theory in liquids have been examined from several experimental and theoretical perspectives that have highlighted the role of nonequilibrium effects. Such nonlinear responses—and their role in light-driven, mechanical–structural phase transitions—originate from the strong coupling of electronic and vibrational degrees of freedom. This effect has also been described for Fröhlich polarons, where the impulsive movement of an electron in the highly nonlinear regime induces persistent coherent phonons (quantized vibrations).

Such nonlinearities pose significant challenges for molecular dynamics (MD) simulations of dissipative systems that are hallmarks of biology, and which reflect the flow of energy under nonequilibrium conditions. The breakdown of linear response theory may thus be closely related to the deviations observed from ergodic behavior in systems of biological relevance. Fluorescence upconversion experiments in the ultraviolet, combined with nonequilibrium MD simulations, have observed deviations from the linear response approximation for the relaxation dynamics of photoexcited tryptophan in water. It has also been demonstrated that metabolic activities drive the biological milieu toward non-ergodicity far from thermodynamic equilibrium, resulting in increased cytoplasmic fluidization that allows larger components to escape their local environment anomalously and explore larger regions of the cellular compartment. Works since 2018 [1,2,3] have predicted, observed, and simulated the emergence and evolution of terahertz-scale phonon coherence in optically driven, out-of-equilibrium proteins in ionic solutions. These “phonon condensates” emerge from a dynamic interplay among physical degrees of freedom in the protein, water, and ions in the solution, and they are intimately related to long-range electrodynamic behaviors in aqueous systems.

Indeed, there is a long-studied relationship between coherent states and fractal self-similarity across physical systems. Since at least the time of Schrödinger (1920s), and more fully developed in the quantum theory of light through seminal works by Bargmann, Segal, Glauber, Sudarshan, Schwinger, and others in the 1960s, a dynamic phase coherence has been proposed between matter coupled to electromagnetic fields. In the quintessential example of the laser, the pumping of a crystal with the appropriate quantum electronic transitions, nonlinearly coupled to its dissipative environment, creates a physical scenario where “population inversion” can occur, violating the thermal equipartition of energy due to the highly driven, nonequilibrated context. This in turn results in a redistribution of energy into the preferred mode(s), which can be “squeezed” in the corresponding space of conjugate, non-commuting observables *A* and *B*, according to the generalized Heisenberg uncertainty relation $\Delta A\;\Delta B\geq \frac{1}{2i}\left[\hat{A},\hat{B}\right]$, where $\hat{A}$ and $\hat{B}$ are the quantum operators corresponding to the observables. Since the 1980s, Vitiello et al. have applied these insights to understanding the emergence of fractal structures via the spontaneous symmetry breaking of coherent states in dissipative environments, which are characteristic of crystals, ferromagnets, superconductors, and biological systems.

In contemporaneous development, spintronic architectures and electron vortex beam setups have been buttressed by advancements in theory on angular momentum conversion between spin and orbital types [4]. Careful analyses [5,6] have shown that electron vortices carry “intrinsic” orbital angular momentum that behaves similarly to the *spin* of a massless particle, even in the non-relativistic limit. In the framework of quantum field theory (QFT), we have over the last five years computed the effects of a static magnetic field on free electron systems [7,8,9]. As with those treatments, the spin $\vec{S}=\left(S_{1},S_{2},S_{3}\right)=\left(S^{23},S^{31},S^{12}\right)$ can be written as follows in terms of the fermionic fields, using ψ(x) as a function of the four-vector $x=(x^{0},\vec{x})$:

(1) $$S^{ab}=\int dx\;\psi^{†}\left(x\right)\;\frac{1}{2}\sigma^{ab}\;\psi \left(x\right)=\int dx\;\psi^{†}\left(x\right)\;\frac{i}{2}\gamma^{a}\gamma^{b}\;\psi \left(x\right).$$

Here, $\gamma^{\mu }=\left(\gamma^{0},\vec{\gamma }\right)$ defines the conventional Dirac matrices. The Weyl (chiral) basis is used in the presentation that follows.

The work described here is motivated by the rich history in condensed matter and particle physics of extending quantum mechanical results by the application of QFT. Considering the field nature of fundamental particles in complex systems can produce drastically different theoretical predictions that may elucidate exotic quantum phenomena at larger scales. In particular, our study makes use of the fact that in QFT spin angular momentum is precisely defined at the outset as a function of the quantum fields, rather than arising in quantum mechanics as an ad hoc addition to the orbital angular momentum (i.e., $\vec{J}=\vec{L}+\vec{S}$). It is important to note that the calculation of infrared, Raman, and terahertz absorption spectra from dipole-dipole correlation functions is contingent upon their derivation in quantum mechanics from first-order perturbation theory (e.g., via Fermi’s golden rule). This work thus provides the foundation for identifying additional terms needed from QFT for the generation of more accurate long-wavelength absorption spectra, in particular when Fermi’s golden rule breaks down.

## 2. Preliminary Details

The purpose of the present article is to study the effects of static electric and magnetic potentials on electron energy. Following the approach for a Dirac electron [10] from prior work [9], we begin with the free-electron Hamiltonian, written explicitly with the four electron field components $\psi_{1},\psi_{2},\psi_{3},\psi_{4}$, such that

(2) $$\begin{array}{ccc} H=\int d\vec{x} & & \left[-\frac{i}{2}\bar{\psi }\gamma^{p}\partial_{p}+\frac{i}{2}\left(\partial_{p}\bar{\psi }\right)\gamma^{p}+m_{e}\bar{\psi }\right]\psi \\ =\int d\vec{x} & & \left\{2m_{e}\;ℜ\left[\;\psi_{1}^{*}\psi_{3}+\psi_{2}^{*}\psi_{4}\;\right]\right.-\left.\;ℑ\left[\;\psi_{1}^{*}\partial_{3}\psi_{1}+\psi_{1}^{*}\left(\;\partial_{1}-i\partial_{2}\;\right)\psi_{2}\right.\right.+\left.\left.\psi_{2}^{*}\left(\;\partial_{1}+i\partial_{2}\;\right)\psi_{1}-\psi_{2}^{*}\partial_{3}\psi_{2}\;\right]\right.\\ & & +\left.\;ℑ\left[\;\psi_{3}^{*}\partial_{3}\psi_{3}+\psi_{3}^{*}\left(\;\partial_{1}-i\partial_{2}\;\right)\psi_{4}\right.\right.+\left.\left.\psi_{4}^{*}\left(\;\partial_{1}+i\partial_{2}\;\right)\psi_{3}-\psi_{4}^{*}\partial_{3}\psi_{4}\;\right]\right.\}. \end{array}$$

Implicit summation over the index *p* spans the spatial components only. An electromagnetic potential is introduced by the covariant four-vector $A_{\mu }=\left(A_{0},\vec{A}\right)$, which will modify the free Hamiltonian via the Dirac minimal-coupling prescription $\partial_{\mu }\to \partial_{\mu }-i\left|e\right|A_{\mu }$. Keeping terms to the first order in the components of $A_{\mu }$, the energy shift due to the electromagnetic potentials has been derived [9]:

(3) $$\Delta_{A_{\mu }}H=2\left|e\right|\int d\vec{x}\;\left\{A_{0}\rho_{0}+\vec{A}\cdot \left[ℜ\left(-\psi_{1}^{*}\psi_{2}+\psi_{3}^{*}\psi_{4}\right),ℑ\left(-\psi_{1}^{*}\psi_{2}+\psi_{3}^{*}\psi_{4}\right),\frac{1}{2}\left(-|\psi_{1}{|}^{2}+|\psi_{2}{|}^{2}+|\psi_{3}{|}^{2}-{\left|\psi_{4}\right|}^{2}\right)\right]\right\},$$

where $\rho_{0}=\sum_{j=1}^{4}{\left|\psi_{j}\right|}^{2}$. Further detail on this calculation can be found in the Methods below.

If we transform the spin current components using the $\gamma^{5}\equiv i\gamma^{0}\gamma^{1}\gamma^{2}\gamma^{3}$ and Pauli matrices $\sigma_{p}$, such that

(4) $$s_{p}^{\prime }=s^{\prime ab}=\frac{1}{2}\psi^{†}\gamma^{5}\sigma^{ab}\psi =\frac{1}{2}\psi^{†}\left[\begin{array}{cc} -I_{2} & 0\\ 0 & I_{2} \end{array}\right]\left[\begin{array}{cc} \sigma_{p} & 0\\ 0 & \sigma_{p} \end{array}\right]\psi =\frac{1}{2}\psi^{†}\left[\begin{array}{cc} -\sigma_{p} & 0\\ 0 & \sigma_{p} \end{array}\right]\psi ,$$

and define $s_{0}^{\prime }\equiv \psi^{†}\left(x\right)\;I_{4}\;\psi \left(x\right)=\rho_{0}$, the energy shift expression takes on a particularly suggestive and compact form:

(5) $$\Delta_{A_{\mu }}H=2\left|e\right|\int d\vec{x}\;A_{\mu }s^{\prime \mu }.$$

Because we can use $\gamma^{5}$ to construct the chirality projection operators $\frac{1}{2}\left(I_{4}\pm \gamma^{5}\right)$, Equation (4) constitutes a chiral transformation of the spin operator $\sigma^{ab}$.

Written in this way, one can see that we have derived a QFT effect analogous to the Zeeman shift in quantum mechanics:

(6) $$\Delta_{\vec{A}}H_{Zeeman}=-g_{s}\mu_{B}\vec{B}\cdot \vec{S},$$

where $g_{s}$ is the familiar gyromagnetic factor and $\mu_{B}$, the Bohr magneton, is half the electron charge-to-mass ratio. One can now see the resemblance between Equation (6) above and the vector potential portion of the QFT scalar product in Equation (5), with roughly a replacement of the magnetic field $\vec{B}$ with the magnetic potential $\vec{A}$ and likewise of the spin $\vec{S}$ with the transformed spin current ${\vec{s}}^{\;\;\prime }$. A similar comparison could be made between the energy shift due to the electric potential $A_{0}$ and the Stark effect.

We now want to compute the expectation value of the energy shift in Equation (5) in a specific electron state. We shall separate this energy shift into the two contributions $\Delta_{A_{0}}H$ and $\Delta_{\vec{A}}H$ from the electric and magnetic potentials, respectively. Consider the state with momentum $\vec{k}$ and defined as a linear combination of two spin eigenstates with complex coefficients. Following the definitions and conventions expressed in [9], namely

(7) $$|\Psi (\vec{k})\rangle =\lambda_{+}|↑,\vec{k}\rangle +\lambda_{-}|↓,\vec{k}\rangle ,$$

where the spin eigenstates are given by

(8) $$|↑,\vec{k}\rangle =\sqrt{2E}\;a_{\vec{k}}^{↑\;†}|0\rangle ,\;|↓,\vec{k}\rangle =\sqrt{2E}\;a_{\vec{k}}^{↓\;†}|0\rangle ,$$

with $E=\sqrt{m_{e}^{2}+{\left|\vec{k}\right|}^{2}}$ and with the state normalization and Fourier transforms of the fermionic fields fixed according to the prescriptions of Peskin and Schroeder [11], we are able to compute the energy shift expectation value from the starting expression

(9) ΔAμH=Ψ(k→)ΔAμHΨ(k→)Ψ(k→)Ψ(k→)Ψ(k→)Ψ(k→).

This integration is complicated by the exact functional form of the potentials $A_{\mu }$. To simplify it, these components are approximated by their average values $\langle A_{0}\rangle ,\langle A_{1}\rangle ,\langle A_{2}\rangle ,\langle A_{3}\rangle$ over the integration volume so they can be treated as numbers. The integration volume is fixed by the scale *d* of the experimental apparatus in which the electron interacts with the potentials. To ease notation in the results below, we will drop the average symbols $\langle \ldots \rangle$ for the potentials, which will hereafter be presumed wherever the $A_{\mu }$ appear.

## 3. Results

Each term in the expectation value (9) must be computed between four sets of bra-kets corresponding to the ↑↑,↓↓,↑↓, and ↓↑ configurations. Additional details on the organization and symmetry of these calculations are included in the Methods. The final magnetic energy shift expression for our single-electron state is thus

(10) $$\langle \Delta_{\vec{A}}H\rangle =\frac{|\vec{k}|}{E}\left|e\right|A_{3}.$$

This is a remarkable result: In the fully relativistic treatment, the first-order energy splitting due to the magnetic potentials is completely independent of the spin-state coefficients $\lambda_{\pm }$. Furthermore, by choosing spinor fields corresponding to spin-*z* eigenstates, only the *z* component of the vector potential $\vec{A}$ survives in the expression for the average energy shift, due to symmetrical but cancelling contributions elsewhere (see the Methods). In the ultra-relativistic limit ($E\approx |\vec{k}\;|$), this shift is proportional to the change ($\left|e\right|A_{3}$) in the conjugate momentum in the *z* direction due to the introduction of magnetic potentials.

Choosing $\vec{A}=\frac{1}{2}\vec{B}\times \vec{x}$, and using sample values for a weak magnet of 3 gauss and an apparatus of length d=1 meter, it is found that $\left|\langle \Delta_{\vec{A}}H\rangle \right|≲0.160$ MeV, just slightly over 30% of the rest mass of the electron. This value is more than 20 times the maximum energy shift for slow electrons ($E\approx m_{e}$) with ${\left(1-v^{2}\right)}^{-1/2}=1.001$, for which $\left|\langle \Delta_{\vec{A}}H\rangle \right|$ is about 1.4% of the electron rest mass. As a comparison, the quantum mechanical Zeeman shift for these characteristic values is orders of magnitude smaller ($10^{-8}$ eV), as one might expect for such weak field strengths. Such QFT estimates are thus a reasonable validation of the employed perturbative approach, which retains the magnetic fields to first order.

The expression for the average energy shift due to the electric potential is similar:

(11) $$\langle \Delta_{A_{0}}H\rangle =2\left|e\right|A_{0}.$$

This electric energy shift is similarly independent of the spin-state coefficients but also lacks information on the electron momentum, which can be understood from the nature of the Lorentz force, $\partial_{0}k_{\mu }=e\left(\partial_{\mu }A_{\nu }-\partial_{\nu }A_{\mu }\right)\partial_{0}x^{\nu }$ or in three-vector notation $\vec{F}=e\left(\vec{E}+\vec{v}\times \vec{B}\right)$. Computing the average energy shifts in the non-relativistic limit (NRL) where $\psi_{1},\psi_{2}\gg \psi_{3},\psi_{4}$, we obtain

(12) $$\begin{array}{ccc} \langle \Delta_{A_{0}}H_{NRL}\rangle = & & \left|e\right|A_{0}\left(1-\frac{|\vec{k}|}{E}\frac{|\lambda_{+}{|}^{2}-{\left|\lambda_{-}\right|}^{2}}{|\lambda_{+}{|}^{2}+{\left|\lambda_{-}\right|}^{2}}\right), \end{array}$$

(13) $$\begin{array}{ccc} \langle \Delta_{\vec{A}}H_{NRL}\rangle = & & \frac{-|e|}{|\lambda_{+}{|}^{2}+{\left|\lambda_{-}\right|}^{2}}\left\{\frac{m_{e}}{E}\left[A_{1}ℜ\left(\lambda_{+}^{*}\lambda_{-}\right)+A_{2}ℑ\left(\lambda_{+}^{*}\lambda_{-}\right)\right]+\frac{1}{2}A_{3}\left[|\lambda_{+}{|}^{2}-{\left|\lambda_{-}\right|}^{2}\right]\right\}+\frac{\left|e\right|A_{3}}{2}\frac{|\vec{k}|}{E}. \end{array}$$

Equations (12) and (13) make apparent that, in the NRL, symmetries are broken that require the inclusion of spin-state information ($\lambda_{\pm }$) in the expressions for the average energy shift. Still, it is interesting to note that there exists a fixed term in each of the electric and magnetic shifts in the NRL that is entirely independent of the spin-state coefficients.

In the low-mass limit, a correspondence exists between the physical description for chirality X and that of helicity:

(14) $$X\overset{m_{e}\ll E}{\to }\frac{\vec{S}\cdot \vec{k}}{\left|\;\vec{k}\;\right|},\;X_{k_{z}}=S_{z},$$

where $\vec{S}$ is the spin for a particle with momentum $\vec{k}=\left(0,0,k_{z}\right)$. Assuming the definition for chirality from Equation (14), it is found that the electric energy shift from Equation (12) can be rewritten as

(15) $$\langle \Delta_{A_{0}}H_{NRL}\rangle =\left|e\right|A_{0}\left(1-\frac{2|\vec{k}|}{E}\langle X_{k_{z}}\rangle \right).$$

Thus, this average shift due to the electric potential in the NRL is a maximum for achiral states $\left(\langle X_{k_{z}}\rangle =0\right)$, and attains a maximum value (equal to the fixed term $\left|e\right|A_{0}$) precisely half that of the fully relativistic result shown in Equation (11). Likewise, the fixed term $+\frac{|\vec{k}|}{E}\left|e\right|A_{3}/2$ in the average magnetic shift (13) is precisely half that of the relativistic shift (10).

Putting our calculations for electric and magnetic potentials together, we obtain the fully general result

(16) $$\langle \Delta_{A_{\mu }}H\rangle =\left|e\right|\left(2A_{0}+\frac{|\vec{k}|}{E}A_{3}\right)$$

to first order, and for achiral electron states, we get

(17) $$\langle \Delta_{A_{\mu }}H_{NRL}^{\mathrm{achir}}\rangle =\left|e\right|\left(A_{0}-\frac{m_{e}}{2E}A_{1}+\frac{|\vec{k}|}{2E}A_{3}\right).$$

For a completely polarized right- ($\lambda_{-}=0$) or left-handed ($\lambda_{+}=0$) electron state, $A_{1}$ and $A_{2}$ terms vanish:

(18) $$\langle \Delta_{A_{\mu }}H_{NRL}^{\mathrm{pol}}\rangle =\left|e\right|\left[A_{0}\left(1\mp \frac{|\vec{k}|}{E}\right)+A_{3}\left(\frac{|\vec{k}|}{2E}\mp \frac{1}{2}\right)\right].$$

**Figure 1:**
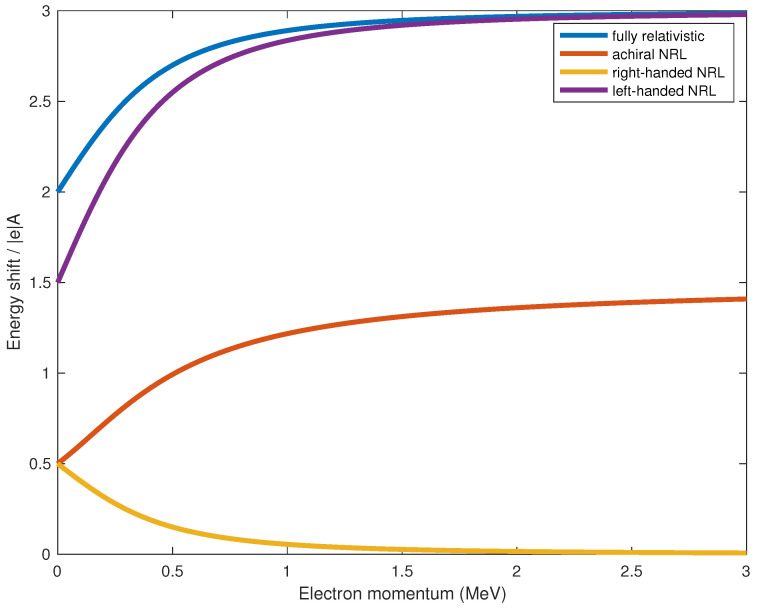
Expectation values of energy shifts due to electromagnetic potentials $A_{\mu }$. The energy shift expectation values, computed in the single-electron state $|\Psi (\vec{k})\rangle =\lambda_{+}|↑,\vec{k}\rangle +\lambda_{-}|↓,\vec{k}\rangle$, have been normalized by $\left|e\right|\bar{A}$, with $\bar{A}=A_{0}=A_{1}=A_{2}=A_{3}$. Dimensionless results for the fully relativistic treatment $\langle \Delta_{A_{\mu }}H\rangle$ (blue), non-relativistic limit (NRL) achiral state $\langle \Delta_{A_{\mu }}H_{NRL}^{\mathrm{achir}}\rangle$ (orange), and NRL completely polarized states $\langle \Delta_{A_{\mu }}H_{NRL}^{\mathrm{pol},\;R}\rangle$ (yellow) and $\langle \Delta_{A_{\mu }}H_{NRL}^{\mathrm{pol},\;L}\rangle$ (purple) are presented as functions of the electron momentum $|\vec{k}|$, in units of MeV. See the Results for further description of these states, in particular Equations (16)–(20).

The difference between these energy shifts,

(19) $$\begin{array}{c} \langle \Delta_{A_{\mu }}\left(H_{NRL}^{\mathrm{pol}}-H_{NRL}^{\mathrm{achir}}\right)\rangle =\left|e\right|\left(\mp \frac{|\vec{k}|}{E}A_{0}+\frac{m_{e}}{2E}A_{1}\mp \frac{1}{2}A_{3}\right) \end{array}$$

and

(20) $$\begin{array}{c} \langle \Delta_{A_{\mu }}\left(H_{NRL}^{\mathrm{pol},L}-H_{NRL}^{\mathrm{pol},R}\right)\rangle =\left|e\right|\left(\frac{2|\vec{k}|}{E}A_{0}+A_{3}\right), \end{array}$$

can be experimentally measured to test the validity of our theory. From Figure 1, we can see that the difference in Equation (20) is larger than that in Equation (19) for all non-zero values of $|\vec{k}|$. Indeed, $\langle \Delta_{\bar{A}}\left(H_{NRL}^{\mathrm{pol},L}-H_{NRL}^{\mathrm{pol},R}\right)\rangle$ is the sum of $\langle \Delta_{\bar{A}}\left(H_{NRL}^{\mathrm{pol},L}-H_{NRL}^{\mathrm{achir}}\right)\rangle$ and $\langle \Delta_{\bar{A}}\left(H_{NRL}^{\mathrm{achir}}-H_{NRL}^{\mathrm{pol},R}\right)\rangle$. This is consistent with what we would expect for achiral states, as they are intermediate between the extremes of completely polarized (right- or left-handed) states.

The energy shifts above are clearly not invariant with respect to the (gauge) potentials but can be transformed in such a way to include only the operationally significant fields. Admittedly, the main results from Equations (10), (11), and (16) only hold true for spatiotemporally constant fields. However, adding a constant vector potential to change only the $A_{1}$ and $A_{2}$ components—which dictate the magnetic field $B_{3}$ along the axis of the critical spin-*z* eigenstates—would not alter Equation (16), in which the average energy splitting is strictly a function of $A_{0}$, $A_{3}$, and electron parameters (cf. discussion between and after Equations (10) and (11)). Described in the Coulomb gauge, and with the constraints above, the average energy shift due to magnetic fields (cf. Equation (10)) is determined to be dependent only on $B_{1}$, $B_{2}$, and the scale of the apparatus over which the fields are effective. If the magnetic field is oriented entirely along the *z*-axis, which in principle can always be done without loss of generality, then the fully relativistic magnetic energy shift vanishes. The non-relativistic magnetic shift from Equation (13), however, does not vanish in this case because $A_{1}=-\frac{1}{2}B_{3}d=-A_{2}$. Thus, the realizable magnetic field functions as the operationally significant quantity in the calculations, and this field (B=∇×A) is not changed by an arbitrary gauge transformation (A→A+∇Λ). The averaging approximation for the potentials described at the end of the Preliminary Details (where the potentials are removed from the integration as numbers) can thus be replaced with the operationally significant fields for each of the resulting energy shifts presented above. Beyond that, it is important to recall the admonition by Aharonov and Bohm, in the closing of their seminal work on the physical effectiveness of electromagnetic potentials [12], that further development of a nonlocal theory is necessary, in which the electron interacts with a field in a finite volume. That is precisely the scenario we have before us.

## 4. Discussion and Conclusions

It has been demonstrated in this article, starting from the Dirac Hamiltonian for a free electron, that a QFT treatment predicts energy shifts induced by magnetic fields acting on the electron spin state that are several orders of magnitude larger than the quantum Zeeman effect. For the fully relativistic treatment, where all four Dirac spinor components are retained, it is observed that the average energy splitting to first order in the potentials is completely independent of the spin-state polarization coefficients. In the NRL, where only the “large” Dirac spinor components are considered, symmetry breaking produces distinctions between achiral and polarized states, and we provide analytical solutions for the different energy shifts that can be experimentally measured.

Our results may be relevant to a range of mesoscopic and macroscopic observables in condensed matter, quantum optics, quantum transport, quantum biology, and a variety of biomedical disciplines. Indeed, though the accurate description of these free or quasi-free electron states is rather complicated, recent studies [13,14,15] suggest that spin polarization enforces symmetry constraints on biorecognition processes between chiral molecules, and that electrons transmitted in charge redistribution processes through chiral molecules are filtered according to spin state and may serve as an allosteric control signal. More generally, it was observed more than three decades ago that a delicate relationship exists between the chirality of enantiomeric crystals formed out of solution, and the low-energy fluctuations that are introduced from exogenous perturbations (e.g., stirring) of the crystallization solution [16]. Diverse spectroscopic approaches [17,18] using ultrafast X-rays and electron vortex beams can be used to probe the molecular chirality of such crystals.

Sensitive dependencies between biological function and the chirality of underlying spin states are apparent with free, as well as bound, electron systems. Many researchers have reported the effects of weak magnetic fields on the rate of adenosine triphosphate (ATP) production [19] and reactive oxidative species (ROS) formation [20] by electron spin flipping in a fashion that preserves quantum coherence. It has also been shown in our group [21] that so-called “palindromic” DNA sequences with a defined chiral mirror symmetry are essential to the synchronization of DNA double-strand breaks, which are catalyzed by a certain class of enzymes used widely in molecular biology, biochemistry, and genomics. Recent theoretical, computational, and experimental work [22,23,24] has demonstrated that the handedness of DNA is reflected and imprinted in the chiral superstructure of its surrounding water matrix.

Consistent with previous works [1,2,3], we are actively pursuing the experimental realization, control, and exploitation of nonequilibrium effects in similarly driven but more complex systems characteristic of biology. The inclusion of magnetic field effects affecting spin degrees of freedom in these driven, nonequilibrium quantum systems will be potentially groundbreaking in augmenting our understanding of how faster life processes at the terahertz scale might influence slower life processes that are commensurate with the functional experience and conscious information processing of whole organisms. Such evidence illuminates the existence of a multiscale, intrinsic structural order connecting electron spin systems to their mesoscopic and macroscopic manifestations, across many orders of magnitude in the physical world.

## 5. Methods

All derivations and calculations were completed by hand, with multiple independent checks. The figure was produced in MATLAB.

To derive Equation (3), we proceed with the following replacements in the integrand of Equation (2), to first order in the potentials:

(21) $$\begin{array}{ccc} & & 2m_{e}ℜ\left(\psi_{1}^{*}\Delta_{A_{\mu }}\psi_{3}+\left(\Delta_{A_{\mu }}\psi_{1}^{*}\right)\psi_{3}+\psi_{2}^{*}\Delta_{A_{\mu }}\psi_{4}+\left(\Delta_{A_{\mu }}\psi_{2}^{*}\right)\psi_{4}\right)\\ & & -ℑ\left[\psi_{1}^{*}\left(-i|e\right|A_{3})\psi_{1}+\psi_{1}^{*}\left(-i|e\right|A_{1}-\left|e\right|A_{2})\psi_{2}+\psi_{2}^{*}\left(-i|e\right|A_{1}+\left|e\right|A_{2})\psi_{1}-\psi_{2}^{*}\left(-i|e\right|A_{3})\psi_{2}\right]\\ & & +ℑ\left[\psi_{3}^{*}\left(-i|e\right|A_{3})\psi_{3}+\psi_{3}^{*}\left(-i|e\right|A_{1}-\left|e\right|A_{2})\psi_{4}+\psi_{4}^{*}\left(-i|e\right|A_{1}+\left|e\right|A_{2})\psi_{3}-\psi_{4}^{*}\left(-i|e\right|A_{3})\psi_{4}\right]. \end{array}$$

To organize the calculations for the expectation value of the change in energy, we consider the numerator of Equation (9), which requires evaluating four bra-kets for each term of the sandwiched operator expression. Starting with $\Delta_{A_{0}}H$, we explicitly evaluate the bra-ket for the ↑↑ configuration:

(22) $$\begin{array}{ccc} & & 2|e|\int d\vec{x}\;A_{0}{\left|\lambda_{+}\right|}^{2}\langle ↑,\vec{k}|\psi_{1}^{*}\psi_{1}+\psi_{2}^{*}\psi_{2}+\psi_{3}^{*}\psi_{3}+\psi_{4}^{*}\psi_{4}|↑,\vec{k}\rangle \\ & & =2|e|A_{0}{\left|\lambda_{+}\right|}^{2}\int d\vec{x}\int \frac{d\vec{p}\;d{\vec{p}}^{\;\prime }}{{\left(2\pi \right)}^{6}}\frac{e^{i\left(\vec{p}-{\vec{p}}^{\prime }\right)\cdot \vec{x}}}{\sqrt{4E_{p}E_{p^{\prime }}}}\langle ↑\left|\sum_{s,s^{\prime }}{a_{p^{\prime }}^{s^{\prime }}}^{†}a_{p}^{s}\left[{u_{1}^{s^{\prime }}\left(p^{\prime }\right)}^{*}u_{1}^{s}\left(p\right)+{u_{2}^{s^{\prime }}\left(p^{\prime }\right)}^{*}u_{2}^{s}\left(p\right)+{u_{3}^{s^{\prime }}\left(p^{\prime }\right)}^{*}u_{3}^{s}\left(p\right)+{u_{4}^{s^{\prime }}\left(p^{\prime }\right)}^{*}u_{4}^{s}\left(p\right)\right]\right|↑\rangle \\ & & =2|e|A_{0}{\left|\lambda_{+}\right|}^{2}\int \frac{d\vec{p}\;d{\vec{p}}^{\;\prime }}{{\left(2\pi \right)}^{6}}\frac{{\left(2\pi \right)}^{3}\delta^{\left(3\right)}\left(\vec{p}-{\vec{p}}^{\prime }\right)}{\sqrt{4E_{p}E_{p^{\prime }}}}\langle 0\left|\left(2E_{k}\right)a_{\vec{k}}^{↑}\sum_{s,s^{\prime }}{a_{p^{\prime }}^{s^{\prime }}}^{†}a_{p}^{s}\left[\;\cdots \;\right]{a_{\vec{k}}^{↑}}^{†}\right|0\rangle \\ & & ={\left(2\pi \right)}^{3}2|e|A_{0}{\left|\lambda_{+}\right|}^{2}\int d\vec{p}\;\frac{2E_{k}}{2E_{p}}\langle 0\left|\delta^{\left(3\right)}\left(\vec{p}-\vec{k}\right)\delta^{↑s^{\prime }}\left[{u_{1}^{s^{\prime }}\left(p\right)}^{*}u_{1}^{s}\left(p\right)+{u_{2}^{s^{\prime }}\left(p\right)}^{*}u_{2}^{s}\left(p\right)+{u_{3}^{s^{\prime }}\left(p\right)}^{*}u_{3}^{s}\left(p\right)+{u_{4}^{s^{\prime }}\left(p\right)}^{*}u_{4}^{s}\left(p\right)\right]\delta^{\left(3\right)}\left(\vec{p}-\vec{k}\right)\delta^{↑s}\right|0\rangle \\ & & ={\left(2\pi \right)}^{3}2|e|A_{0}{\left|\lambda_{+}\right|}^{2}\delta^{\left(3\right)}\left(0\right)\left[{u_{1}^{↑}\left(k\right)}^{*}u_{1}^{↑}\left(k\right)+{u_{2}^{↑}\left(k\right)}^{*}u_{2}^{↑}\left(k\right)+{u_{3}^{↑}\left(k\right)}^{*}u_{3}^{↑}\left(k\right)+{u_{4}^{↑}\left(k\right)}^{*}u_{4}^{↑}\left(k\right)\right]\\ & & ={\left(2\pi \right)}^{3}2|e|A_{0}{\left|\lambda_{+}\right|}^{2}\delta^{\left(3\right)}\left(0\right)\left[\left(E-k_{z}\right)+0+\left(E+k_{z}\right)+0\right]=\begin{array}{c} 4|e|A_{0}E{\left|\lambda_{+}\right|}^{2}{\left(2\pi \right)}^{3}\delta^{\left(3\right)}\left(0\right) \end{array}, \end{array}$$

where in the last line we have employed the use of the spinor fields from [11]. We see that the more general expression for these spinors along a fermion spin-component axis with coordinates θ,ϕ can be derived [11] from the two-component spinors

(23) $$\xi \left(↑\right)=\left(\begin{array}{c} \cos\frac{\theta }{2}\\ e^{i\phi }\sin\frac{\theta }{2} \end{array}\right),\;\xi \left(↓\right)=\left(\begin{array}{c} -e^{-i\phi }\sin\frac{\theta }{2}\\ \cos\frac{\theta }{2} \end{array}\right).$$

By symmetry, we obtain a result similar to the boxed quantity (22) for the ↓↓ configuration, with the replacement $\lambda_{+}\to \lambda_{-}$. We get zero contributions from both opposite-spin configurations. Note that in $\Delta_{A_{0}}H$, the normalization for our spin state Ψ in the denominator of the expectation value precisely cancels the factor of $\left(\right|\lambda_{+}{|}^{2}+|\lambda_{-}{|}^{2})E{\left(2\pi \right)}^{3}\delta^{\left(3\right)}\left(0\right)$ contributed by the same-spin configurations.

Moving to $\Delta_{\vec{A}}H$, we note that there are *zero* contributions from the $A_{1}$ and $A_{2}$ terms, due to precise cancellation of contributions from the opposite-spin configurations, e.g.,

(24) $$\begin{array}{ccc} & & \int d\vec{x}\;\langle ↑,\vec{k}|ℜ\left(-\psi_{1}^{*}\psi_{2}\right)|↓,\vec{k}\rangle =-\int d\vec{x}\;\langle ↑,\vec{k}|ℜ\left(\psi_{3}^{*}\psi_{4}\right)|↓,\vec{k}\rangle =-{\left(2\pi \right)}^{3}\delta^{\left(3\right)}\left(0\right)\frac{m_{e}}{2},\\ & & \int d\vec{x}\;\langle ↑,\vec{k}|ℑ\left(-\psi_{1}^{*}\psi_{2}\right)|↓,\vec{k}\rangle =-\int d\vec{x}\;\langle ↑,\vec{k}|ℑ\left(\psi_{3}^{*}\psi_{4}\right)|↓,\vec{k}\rangle =+{\left(2\pi \right)}^{3}\delta^{\left(3\right)}\left(0\right)\frac{im_{e}}{2}, \end{array}$$

and nothing from the same-spin configurations. By symmetry with the $A_{0}$ bra-kets computed above, we can easily find the $A_{3}$ terms as expressed in the following relations:

(25) $$\begin{array}{c} \int d\vec{x}\langle ↑,\vec{k}|-|\psi_{1}{|}^{2}+|\psi_{2}{|}^{2}+|\psi_{3}{|}^{2}-|\psi_{4}{|}^{2}|↑,\vec{k}\rangle ={\left(2\pi \right)}^{3}\delta^{\left(3\right)}\left(0\right)2|\vec{k}|=\int d\vec{x}\langle ↓,\vec{k}|-|\psi_{1}{|}^{2}+|\psi_{2}{|}^{2}+|\psi_{3}{|}^{2}-{\left|\psi_{4}\right|}^{2}|↓,\vec{k}\rangle . \end{array}$$

Therefore the total contributions to $\Delta_{\vec{A}}H$ in our spin state Ψ *all* come from the $A_{3}$ terms, with a similar cancellation of a factor of $\left(\right|\lambda_{+}{|}^{2}+|\lambda_{-}{|}^{2}){\left(2\pi \right)}^{3}\delta^{\left(3\right)}\left(0\right)$ by the fixed normalization in the denominator of the expectation value.

## Data Availability

Not applicable.
